# A Comparative Analysis and Phylogenetic Relationship of the Chloroplast Genome Sequences of *Illicium verum* and *Illicium difengpi*

**DOI:** 10.3390/genes16030321

**Published:** 2025-03-08

**Authors:** Suqin Guo, Xiqun Wu, Feng Peng, Kun Zhang, Suren Rao Sooranna, Guiyu Tan

**Affiliations:** 1 School of Pharmacy and Food Engineering, Wuyi University, Jiangmen 529020, China; guosuqin074910@163.com (S.G.); 18276826337@163.com (X.W.); 2 Key Laboratory of High-Quality Formation and Utilization of Dao-di Herbs, Guangxi Botanical Garden of Medicinal Plants, Nanning 530023, China; beangfeng@163.com (F.P.); zhkun_lc@126.com (K.Z.); 3 Department of Metabolism, Digestion and Reproduction, Faculty of Medicine, Imperial College London, London SW10 9NH, UK; s.sooranna@imperial.ac.uk

**Keywords:** *Illicium verum*, *Illicium difengpi*, chloroplast genome, phylogenetic relationship

## Abstract

**Background/Objectives:** *Illicium verum* Hook. f. and *Illicium difengpi* K. I. B.et K. I. M. are two important medicinal plants which grow in the mountainous areas of Guangxi, China. Their similar morphological characteristics frequently lead to their misidentification. Chloroplast genome (cp)-based barcode technology has been used to effectively identify two closely related species; however, at present, there is no systematic comparative study of the cp genome sequences between *I. verum* and *I. difengpi*. **Methods**: Here, the cp genomes of the two plants were sequenced and analyzed. **Results**: The cp genome sizes were 142,689 and 142,689 bp for *I. verum* and *I. difengpi*, respectively. Each of the cp genomes annotated 122 genes, with 79 protein coding genes, 8 ribosomal RNA genes, and 35 transfer RNA genes. Amino acid frequencies of 1.17–10.19% (*I. verum*) and 1.18–10.17% (*I. difengpi*) were found in the coding genes. There were also 104 and 96 SSRs as well as 26 and 25 long repeats identified in *I. verum* and *I. difengpi*, respectively, among which the most common were A/T base repeats. Both cp genomes had SSC/IRa junctions located in gene *ycf1-trnN.* The *ycf1* and *trnL-trnV-rps7* genes were positioned at the IRb/SSC and LSC/IR boundaries, respectively. A phylogenetic relationship was constructed and the two species were fully nested within the genus *Illicium*. **Conclusions**: The comparative cp genomes of *I. verum* and *I. difengpi* are presented in this study, and this provides valuable phylogenetic information for subsequent molecular marker development and research of *I. verum* and *I. difengpi*.

## 1. Introduction

The classification of *Illicium* L. is controversial. It was recognized as an independent family of Illiciaceae according to the APG II system [1] and it had merged with Schiasndraceae since the APG III [2]. At present, 42 *Illicium* species are recognized, and their similar morphological characteristics frequently lead to serious misidentifications. The *I. verum* Hook. f. and *I. difengpi* K. I. B.et K. I. M. are two important medicinal plants which are found in the mountainous areas of Guangxi, China. This region supplies 80% of the relative production of *I. verum* and *I. difengpi* for the global market [3]. Both these plants grow and thrive in mountainous environments and they provide essential habitats and food sources for various organisms within the ecosystem. Their root systems contribute to soil stabilization and reduce soil erosion, thereby helping to maintain soil structure and fertility. The volatile compounds present in the fruits and leaves of *I. verum* exhibit a natural repellent effect against certain pests, thereby minimizing damage to other plants [4]. *I. difengpi* possesses significant drought resistance and can adapt to the bare and semi-bare rocky environments of karst stone mountains [5]. It plays a crucial role in the ecological restoration of karst landscapes. Both the plants are embodied in the Chinese pharmacopeia and have been extensively utilized in traditional herbal medicine for centuries. *I. verum* is one of the most well-known plants of *Illicium*, and its fruits have antimicrobial, antioxidant and anti-diabetes activities [6]. Shikimic acid, one of most important compounds in *I. verum*, is used as a drug to enable the body to fight against the influenza and flu viruses. It is also used as a raw material in spices and cosmetics. *I. difengpi* has remarkable anti-inflammatory activity [7,8] and it has curative effects in the treatment of rheumatic arthralgia, making it the primary raw material for various traditional Chinese medicines, including Guilong ointment and medicinal wine. The two plants with morphological similarities have different medical properties. As two commonly used medicine sources in the region, their confused usage potentially delays their use for appropriate medical treatments. To distinguish between the plants, Huang et al. [9] indicated that the fruits of *I. verum* taste sweet, while those of *I. difengpi* were sour. With respect to their chemical composition, the volatile oil content of *I. verum* (5.9–13.5%) was determined to be significantly higher than that of *I. difengpi* (0.6–1.0%) [10]. Comprehensive comparative analysis could provide a foundation for the establishment of efficient identification methods in the future.

Nowadays, chloroplast (cp) genome technology has emerged as a powerful tool for distinguishing closely related plant species. The cp genomes consist of circular DNA molecules that typically range from 120 to 160 kb in length. These genomes exhibit a quadripartite structure, which comprises two inverted repeats (IRs) and a large-single-copy (LSC) and small-single-copy (SSC) region [11]. The intraspecific cp genome maintains remarkable conservation in their structure, gene composition, and genomic arrangement. In contrast, the interspecific cp genome displays a comparatively high degree of genetic variation [12]. The differential cp genomic DNA sequences can be used to identify species rapidly and efficiently and, therefore, this has become a hotspot of molecular identification research. The cp genomes of *I. verum* [13], *I. anisatum* [14], *I. simonsii* Maxim [15], *I. henryi*, and *I. oligandrum* [16] have previously been sequenced and subjected to dynamic evolution and phylogenomic analysis. In addition, Liu et al. [17] have used a DNA barcode of *psbA-trnH*, which are two adjacent genes at the LSC and IRB boundary, for discrimination between *I. verum* and its adulterants in trade. Recently, mini-barcodes were used to distinguish *I. difengpi* from other three species of *Illicium* [18]. The results demonstrated the effectiveness of DNA mini-barcodes of cp genomes to identity the authenticity of the *I. difengpi*.

Although the characteristic sequences within the cp genomes have already been used to distinguish some *Illicium* species, systematic comparative studies of this type between *I. verum* and *I. difengpi* are still lacking. Here, the cp genomes were sequenced and characterized for *I. verum* and *I. difengpi* and these were analyzed against the published data of other *Illicium* species, to study the genetic relationships and plant identification. These results will allow the effective identification of the two plants.

## 2. Materials and Methods

### 2.1. Plant Materials

Leaves were gathered at Guangxi Botanical Garden of Medicinal Plants, Nanning, China (E 108°22′01″, N 22°51′26″). The two plant samples were identified and subsequently archived in the herbarium of this institute.

### 2.2. Sequencing and Genome Assembly

Density gradient centrifugation was used to separate the chloroplasts from the leaves of *I. verum* and *I. difengpi*, respectively. These were digested with DNase I to remove contamination from genomic DNA (Promega, Madison, WI, USA). The complete cp genome sequences were obtained through the application of long-read (PacBio Sequel II, Menlo Park, CA, USA) and short-read (Illumina, San Diego, CA, USA) sequencing platforms. Exceeding 4.0 Gb data for each sample was successfully generated. The quality assessment of the short read was conducted via FastQC, followed by read trimming through Trimmomatic. The read filter included the remove adapter sequences and trimmed non-AGCT bases at the 5′ ends. In addition, reads with quality scores below Q20, reads with more than 10% bases as N, and fragments shorter than 75 bp were excluded. The long-read sequencing data underwent base-calling and barcode de-multiplexing via Albacore v2.1.7, followed by conversion to FASTA format utilizing Samtools http://www.htslib.org/doc/samtools.html (accessed on 11 July 2023). The potential cp contigs were identified through alignment against the cp protein-coding genes ftp://ftp.ncbi.nih.gov/refseq/release/plastid/ (accessed on 11 July 2023) using BLAST v2.8.1+. Long cp reads were captured by mapping with BLASR v5.1. Subsequently, short and long reads were assembled with GetOrganelle v1.6.4 and Canu v2.1.1, respectively.

### 2.3. Gene Annotation

The protein coding genes, transfer RNA (tRNA) genes, and ribosome RNA (rRNA) genes were annotated using the GeSeq tool. Functional annotations were conducted through sequence-similarity Blast searches, employing a standard cut-off of 10^−5^. The NCBI Nr, Swiss-Prot, COGs, KEGG, and GO databases were used in this step. The OGDRAW tool was then applied to generate circular maps of the cp genomes.

### 2.4. Comparison and Differential Analysis of Cp Genomes

The Shuffle-LAGAN mode of mVISTA software http://genome.lbl.gov/vista/mvista/submit.shtml (accessed on 11 July 2023) was employed for comparing genome structures. The published *I. verum* cp genome served as the reference. The cp genome nucleotide diversity was then analyzed by DnaSP v5.10 software. The relative synonymous codon usage (RSCU) for the protein-coding genes of *I. verum* and *I. difengpi* cp genomes were measured by CUSP (EMBOSS v6.6.0.0). Tandem repeats and SSRs in the cp genomes were detected by Tandem Repeats Finder and MIcroSAtellite (MISA), respectively. Their size and location were determined via REPuter software http://bibiserv.techfak.uni-bielefeld.de/reputer/ (accessed on 11 July 2023). The IRScope software package https://irscope.shinyapps.io/irapp/ (accessed on 20 March 2024) was applied to visualize the contraction and expansion of IR regions among the LSC, IRb, SSC, and IRa regions. The Phylogenetic relationship was established with the maximum likelihood (ML) method by software PhyML v3.0.

## 3. Results

### 3.1. Chloroplast Genome Structure and Composition

**Figure 1:**
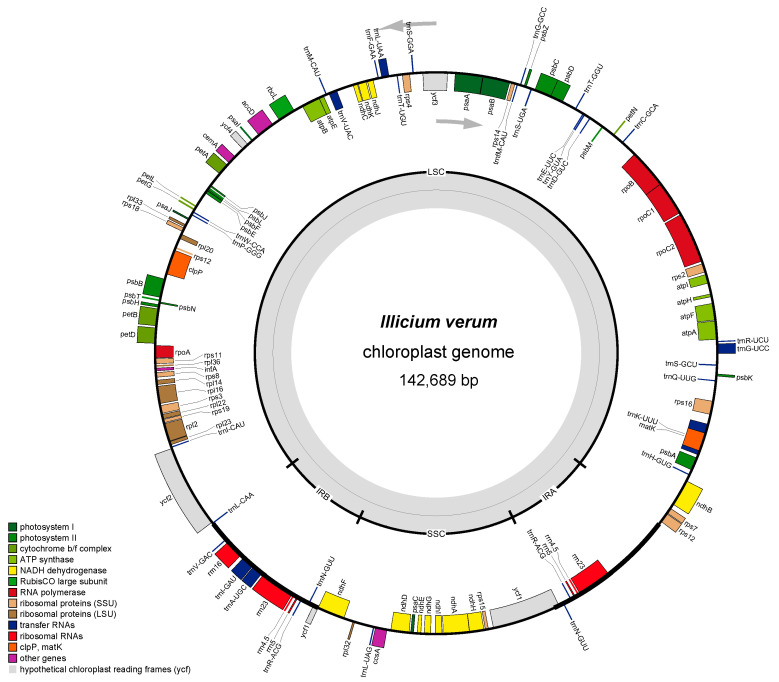
A diagrammatic representation of the *I. verum* chloroplast genome. The outer circle comprises genes of different functional groups. The inner circle indicates the conserved quadripartite structure.

A total of 37,253,748 reads for *I. verum* resulted from the Illumina paired-end sequencing and after trimming, 5511.6 Mb of clean data were acquired. The cp genome length was 142,689 bp. The LSC, SSC, and IR regions were 101,100, 19,687, and 10,949 bp, with GC content of 37.96, 34.16, and 49.06%, respectively (Table S1). A total of 122 genes were annotated, with an average length of 871 bp. There were 79 protein coding genes, 8 rRNA genes, and 35 tRNA genes. The coding gene length was 68,790 bp, which is 48.21% of the cp genome (Figure 1).

**Figure 2:**
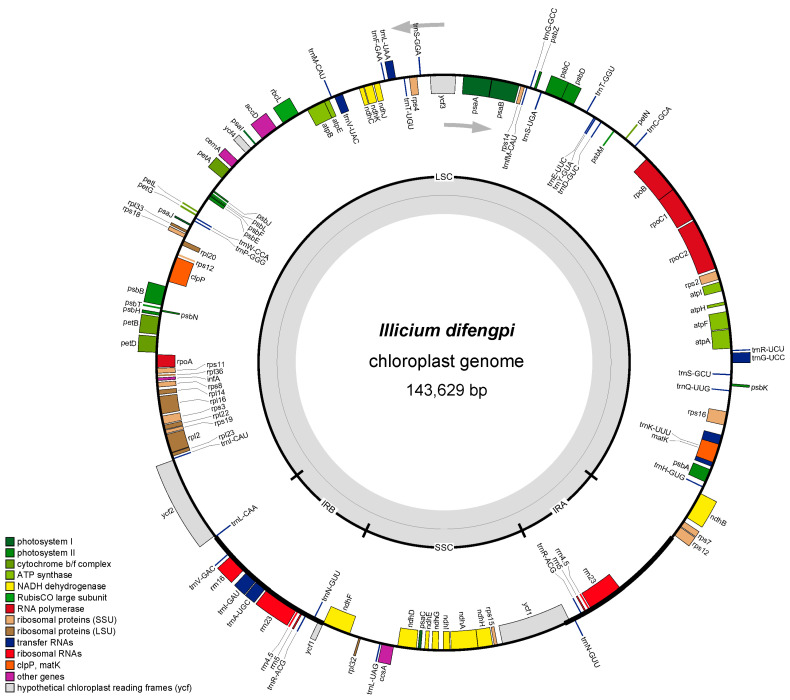
A diagrammatic representation of the *I. difengpi* chloroplast genome. The outer circle comprises genes of different functional groups. The inner circle indicates the conserved quadripartite structure.

The cp genome of *I. difengpi* was also obtained (Figure 2) consisting of 32,141,426 reads and 4743.4 Mb of clean data. The cp genome was 143,629 bp, with the LSC, SSC, and IR regions being 101,450, 20,237, and 10,971 bp in length, respectively. The GC contents of the IR, SSC, and LSC areas were 49.06, 33.91, and 37.98%, respectively (Table S1). The total, encoded, rRNA, and tRNA gene numbers of *I. difengpi* were highly consistent with those of *I. verum*. A total of 47.97% of the cp genomes of *I. difengpi* comprised the coding genes, whose average and total lengths were 872 and 68,898 bp, respectively.

The annotated genes of *I. verum* and *I. difengpi* were generally identical, and these included photosynthetic, self-replicating and functional unknown *ycf* genes. Nine gene duplications were seen in the IR regions, with one coding gene (*ycf1*), four rRNA genes (*rrn4.5*, *rrn5*, *rrn16*, and *rrn23*), and four tRNA genes (*trnA-UGC*, *trnI-GAU*, *trnN-GUU*, and *trnV-GAC*). Furthermore, ten coding genes and six tRNA genes contained single introns and *ycf3* and *clpP* contained two introns (Table 1).

### 3.2. Codon Bias Analysis

There were sixty-one types of codons encoding twenty amino acids found for each of the two plants, with six codons encoding arginine, leucine, and serine and four codons encoding alanine, glycine, proline, threonine, and valine. There were also three codons encoding isoleucine, two codons encoding asparagine, aspartic, cysteine, glutamine, glutamic acid, histidine, lysine, phenylalanine, and tyrosine, and one codon encoding methionine and tryptophan. Leucine exhibited the highest frequencies of 10.15% (*I. verum*) and 10.17% (*I. difengpi*), while Cysteine was the lowest with 1.16% (*I. verum*) and 1.18% (*I. difengpi*) in coding genes. Codons with an RSCU value exceeding 1.0 could be considered as preferred codons. Notably, methionine and tryptophan exhibited an RSCU value of 1.0, since each was encoded by a single codon. There were 30 codons out of 61 that demonstrated RSCU values greater than 1.0, with a significant proportion (28/30) of these preferred codons terminating in either A or T. Furthermore, arginine, alanine, and leucine consistently emerged as amino acids with the highest RSCU values. Specifically, the codons AGA, GCT, and TTA showed usage bias for the three amino acids, with 1.86, 1.76, and 1.73 for *I. verum* and 1.87, 1.79, and 1.73 for *I. difengpi*, respectively (Table S2).

### 3.3. Simple Sequence Repeats (SSRs) and Long Repeats

The MISA was applied to analyze the SSRs in this study (Table S3). A total of 104 and 96 SSRs were determined in *I. verum* and *I. difengpi* cp genomes, respectively. The SSRs were predominantly distributed in LSC, with subsequent occurrences in the SSC and IR. There were 6, 81, and 17 SSRs in the IR, LSC, and SSC of *I. verum* cp genome, respectively. As for *I. difengpi*, the numbers were 8 (IR), 70 (LSC), and 18 (SSC). The number of SSRs in the coding regions was 16 in *I. verum* and 15 in *I. difengpi*. Among these, mononucleotide repeats were the most frequently observed, with 80 and 74 found, respectively. These were followed by dinucleotide (8 and 7), tetranucleotide (5 and 4), trinucleotide (9 and 8), and pentanucleotide (2 and 1) repeats, respectively. There were no hexanucleotide repeats in the *I. verum* cp genome, but there were two in *I. difengpi*. Additionally, the majority of the mononucleotide SSRs were A/T repeats. They represented 55 (*I. verum*) and 59% (*I. difengpi*) of cp genomes, followed by other repeats, showing 23 (*I. verum*) and 19% (*I. difengpi*), and both of the remaining repeat types were present at 22%.

In this study, 27 long repeats that consisted of 12 forward, 11 palindromic, and 4 reverse repeats were determined in *I. verum* using the REPuter software. Most sequences were between 30 and 34 bp (19) in length, followed by 35–39 bp (4). The lengths of 40–44, 45–49, 55–59, and ≥70 bp segments all had only one long repeat. For *I. difengpi*, 26 long repeats that consisted of 12 palindromic repeats, 8 forward repeats, and 6 reverse repeats were detected. Among all of the long repeats, most sequences were between 30 and 34 bp (14) in length, followed by 35–39 (8) and 45–49 bp (2). The long repeats of 40–44 bp and ≥70 bp both had the lowest number. There was no complement repeat matched in the two plants.

### 3.4. IR Expansion and Contraction

**Figure 3:**
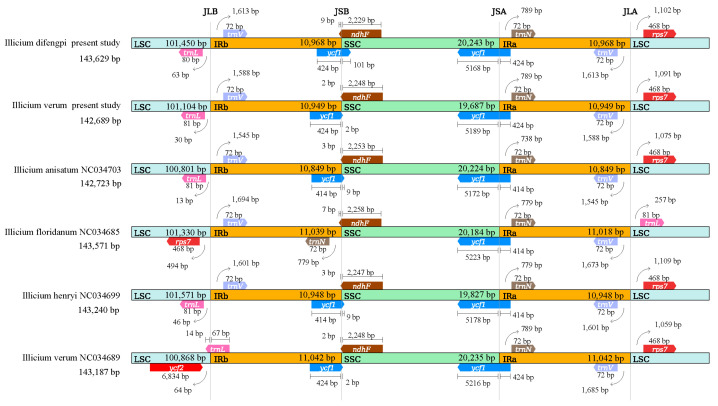
A comparison of the borders of large-single-copy (LSC), small-single-copy (SSC), and inverted-repeat (IR) regions. The JLB, JSB, JSA, and JLA lines indicate the borders between the LSC and IRb, IRb and SSC, SSC and IRa, and IRa and LSC regions, respectively.

The positions of IR boundaries and adjacent genes of *I. verum* and *I. difengpi*, as well as three other *Illicium* species, were compared (Figure 3). The IR lengths were relatively consistent among all of the *Illicium* species. *Illcium anisatum* had the shortest IR length (21,698 bp), while *Illicium floridanum* had the longest (22,057 bp). All of the *Illicium* species’ cp genomes had SSC/IRa junctions (JSA) located in gene *ycf1-trnN*, with *ycf1* extending 414–424 bp into the IRa regions. The *ycf1* gene exhibited duplication; it was positioned at the IRb/SSC (JSB) boundary and extended 2–101 bp into the SSC region, but this did not occur in *I. floridanum*. The *trnL-trnV-rps7* in all of these species was positioned at the intersections of the LSC/IR. The *trnL* and *rps7* were entirely in the LSC regions in the five species, being 81 and 468 bp in length, respectively. The *trnV* gene was entirely in the IR region and it exhibited duplication in all cp genomes with a gene length of 72 bp. The *trnN* gene was only duplicated and positioned at the IRb of *I. floridanum*. Compared to the other four plants, there was inversion in the LSC sequence of *I. floridanum*.

### 3.5. Phylogenetic Analysis

**Figure 4:**
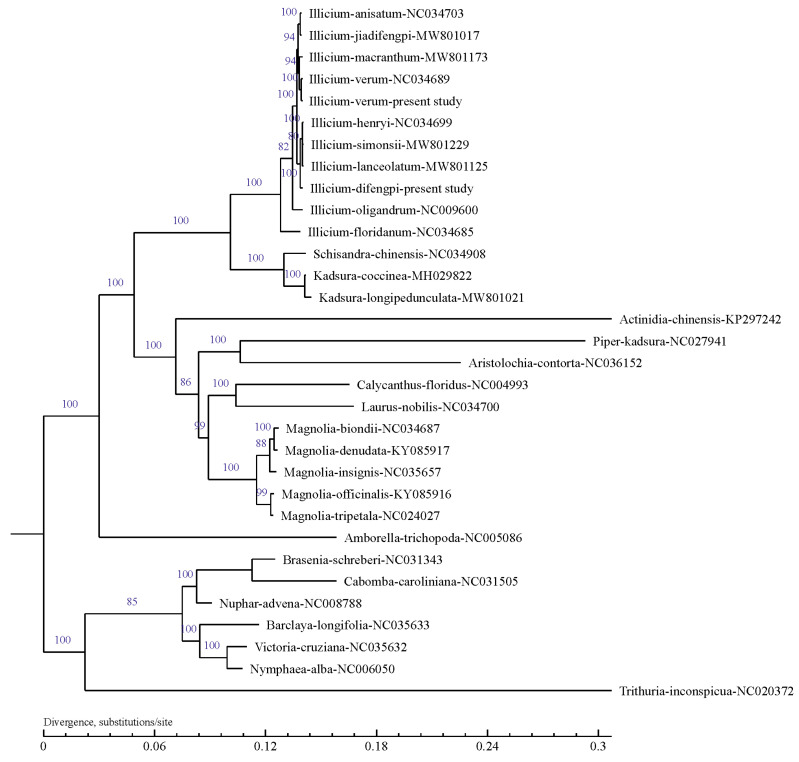
A phylogenetic tree constructed with the chloroplast genomes of *I. verum* and *I. difengpi*. The values above the branches indicate the maximum likelihood bootstrap values.

A phylogenetic tree was established in order to evaluate the phylogenetic relationship of *I. verum* and *I. difengpi* using the ML method (Figure 4). Most nodal support values were high and this was verified with ML bootstrap support values (ML BSs) >80. The *Illicium* species were clustered together as a monophyletic clade with the ML BS values of 100%, and they formed a group with the sister clade consisting of *Schisandra chinensis* and its two other sections (*Kadsura coccinea* and *Kadsura longipedunculata*). However, the *Illicium* clade was located far from the clade consisting of *Magnolia biondii*, *Magnolia denudate*, *Magnolia insignis*, *Magnolia officinalis*, and *Magnolia tripetala*. In addition, *I. verum* and *I. difengpi* were fully nested within *Illicium* with an ML BS value of 100%. The reported *I. verum* cp genome (NCBI code: NC034689) was found to be clustered together with the present cp genome (ML BS: 100%), and *I. difengpi* was placed in a sister group.

## 4. Discussion

The significant degree of morphological similarities among the *Illicium* species makes it challenging to distinguish between *I. verum* and *I. difengpi* and this is essential for their use as traditional medicines. The cp genome-based barcode technology of *I. verum* and *I. difengpi* has been used to solve this issue. The genome sizes of *I. verum* and *I. difengpi* were determined to be 142,689 and 143,629 bp, respectively, which are similar to the previously published data: 143,187 bp for *I. verum* (NC_034689.1) and 143,629 bp for *I. difengpi* (NC_063671.1). Although the cp genome sizes varied among the *Illicium* species, there were only minor differences (142,689–148,553 bp), implying that they are relatively conserved. Additionally, both of the cp genomes exhibited a four-part structure. The structures and gene orders displayed significant conservation, aligning with the previously published *Illicium* cp genomes [13,14,15]. The GC content may significantly influence the function of the cp genome through various aspects, including DNA stability, transcription and translation efficiency, gene expression regulation, genomic structure, and evolutionary adaptability. The two cp genomes had similar GC content, suggesting a high degree of genomic similarity between them. Notably, the GC content of the IR region, 49.06% for both of the cp genomes, was higher than that in the other two regions: 37.96 and 37.98% for the LSC, as well as 34.14 and 33.91% of the SSC. It may be related to the several rRNA genes within the IR regions. When compared with other plants, the GC contents of the IR, LSC, and SSC in both cp genomes were within a normal range.

The present phylogenetic tree showed that the *Illicium* plants formed a monophyly and they were closely related to the *Schisandraceae*, which included the genera *Schisandra* and *Kadsura*. This conclusion is in line with earlier phylogenetic studies [19]. Morris et al. [20] took biogeographic considerations into account and provided a revised sectional classification of the *Illicium* genus with two sections: Old World species (distributed across southeastern Asia) and New World species (distributed in southeastern U.S., Mexico, Cuba, and Hispaniola). In the present work, the *I. floridanum* formed a single clade with the other *Illicium* species being projected as a sister clade, and this was also found in previous studies. Fossil records demonstrate that the genus *Illicium* had a more extensive geographical distribution during earlier geological periods, and its modern distribution pattern (discontinuous distribution in the Old World and the New World) may be related to continental drift following the separation of the ancient landmasses. In Asia, the genus *Illicium* is distributed primarily in mountain valleys and along the sides of streams, where they participate in the creation of a variety of microclimates and microhabitats. The karst mountains in Guangxi have developed unique hydrothermal conditions through erosion, potentially leading to the emergence of local endemic species, such as *I. difenpi*. The mountains have impeded genetic exchange between East Asian and Southeast Asian populations, thereby facilitating the radiation evolution of *Illicium* in East Asia and further reinforcing the accumulation of genetic diversity under conditions of geographic isolation. The pollinators of the genus *Illicium* are primarily small pests, which have a limited flight range [21]. Combined with a restricted ballistic dispersal method [22], all of the members of the genus *Illicium* exhibit very limited geographical distribution and phylogenetic constraints. However, the color of the petals and fragrance may attract specific insects for pollination. The differences in pollinator communities across different geographical regions may drive the diversification of flower color and morphology of the plants.

With abundant loci associated with polymorphisms in the cp genomes of the genus *Illicium*, they are often used as DNA barcodes to identify precise species, and this could facilitate better resource usage of the genus *Illicium*. As previously reported, the *psbA-trnH* [15], *atpF-atpH*, and *ndhF-rpl32* [6] regions could be applied to identify *I. verum* and other similar plants. Further, the cp mini-barcodes, *trnL-trnF*, *trnF-ndhJ*, *ycf1-ndhF*, and *rpl32-trnL*, were proposed as powerful tools to distinguish *I. difengpi* from other *Illicium* plants [18]. For the family Schisandraceae, *ITS*+*trnH*-*psbA*+*matK*+*rbcL* was identified as the optimal DNA barcode for discriminating *Schisandra* and *Kadsura* plants, while *ITS*+*trnH*-*psbA* proved to be an effective barcode for differentiation within *Illicium* plants. The most appropriate DNA barcode for taxonomic discrimination may vary between different taxonomic levels [23]. In this study, 104 and 96 SSRs were detected in *I. verum* and *I. difengpi*, respectively. They were at relatively high levels compared to 43 SSRs in *K. coccinea*, 74 SSRs in *S. chinensis*, and 100 SSRs in *I. oligandrum* [19]. This phenomenon could lead to the faster evolution of cp genomes in the region, thereby allowing for adaptation to environmental changes. The SSR distribution might also affect the function of the cp genomes by regulating gene expression and genomic stability. Due to the high repeatability and ease of amplification, the SSRs could be potential molecular markers. A cp genome that is characterized by a high copy number may enable more rapid and precise detection methods. Consequently, the utilization of SSRs within cp genomes, particularly for species identification, presents considerable potential. Therefore, effective species differentiation may be achieved by analyzing the SSR sequences and their repeat numbers across various plants. In the field of traditional Chinese medicine, the utilization of differentiated SSRs as well as their precise combinations will help to ensure the purity and quality of the medicinal products.

## 5. Conclusions

In this research, the cp genomes of *I. verum* and *I. difengpi* were sequenced and assembled. Their structural features, genome sizes, GC content, and repeats were found to exhibit remarkable similarity to those of most *Illicium* plants. Furthermore, the phylogenetic relationship of *I. verum* and *I. difengpi* was evaluated. These findings not only expand upon the cp genomic resources for *Illicium* but they will also offer clues for understanding the evolutionary of *Illicium*.

## Figures and Tables

**Table 1:** The chloroplast genomic genes of *I. verum* and *I. difengpi*.

Category	Gene Groups	Gene Names
Photosynthesis	Subunits of photosystem I	*psaA*, *psaB*, *psaC*, *psaI*, *psaJ*
	Subunits of photosystem II	*psbA*, *psbB*, *psbC*, *psbD*, *psbE*, *psbF*, *psbH*, *psbJ*, *psbK*, *psbL*, *psbM*, *psbN*, *psbT*, *psbZ*
	Subunits of NADH dehydrogenase	*ndhA* ^a^, *ndhB* ^a^, *ndhC*, *ndhD*, *ndhE*, *ndhF*, *ndhG*, *ndhH*, *ndhI*, *ndhJ*, *ndhK*
	Subunits of cytochrome b/f complex	*petA*, *petB* ^a^, *petD* ^a^, *petG*, *petL*, *petN*
	Subunits of ATP synthase	*atpA*, *atpB*, *atpE*, *atpF* ^a^, *atpH*, *atpI*
	Large subunit of Rubisco	*rbcL*
Self-replication	Large subunits of ribosome	*rpl14*, *rpl16* ^a^, *rpl2* ^a^, *rpl20*, *rpl22*, *rpl23*, *rpl32*, *rpl33*, *rpl36*
	Small subunits of ribosome	*rps11*, *rps12* ^a^, *rps14*, *rps15*, *rps16* ^a^, *rps18*, *rps19*, *rps2*, *rps3*, *rps4*, *rps7*, *rps8*
	DNA-dependent RNA polymerase	*rpoA*, *rpoB*, *rpoC1* ^a^, *rpoC2*
	Ribosomal RNAs	*rrn16* (×2), *rrn23* (×2), *rrn4.5* (×2), *rrn5* (×2)
	Transfer RNAs	*trnA-UGC* (×2) ^a^, *trnC-GCA*, *trnD-GUC*, *trnE-UUC*, *trnF-GAA*, *trnG-GCC*, *trnG-UCC* ^a^, *trnH-GUG*, *trnI-CAU*, *trnI-GAU* (×2) ^a^, *trnK-UUU* ^a^, *trnL-CAA*, *trnL-UAA* ^a^, *trnL-UAG*, *trnM-CAU*, *trnN-GUU* (×2), *trnP-GGG*, *trnQ-UUG*, *trnR-ACG*, *trnR-ACG*, *trnR-UCU*, *trnS-GCU*, *trnS-GGA*, *trnS-UGA*, *trnT-GGU*, *trnT-UGU*, *trnV-GAC* (×2), *trnV-UAC* ^a^, *trnW-CCA*, *trnY-GUA*, *trnfM-CAU*
Other genes	Maturase	*matK*
	Protease	*clpP* ^b^
	Envelope membrane protein	*cemA*
	Acetyl-CoA carboxylase	*accD*
	C-type cytochrome synthesis gene	*ccsA*
	Translation initiation factor	*infA*
	Protochlorophyllide reductase subunit	*--*
Genes of unknown	Proteins of unknown function	*ycf1* (×2), *ycf2*, *ycf3* ^b^, *ycf4*

^a^ Gene containing single intron; ^b^ gene containing two introns; (×2) gene containing two copies.

## Data Availability

The original contributions presented in the study are included in the article/Supplementary Material, further inquiries can be directed to the corresponding author.

## Supplementary Materials

The following supporting information can be downloaded at https://www.mdpi.com/article/10.3390/genes16030321/s1: Table S1: Characteristics of the complete chloroplast genomes of *I. verum* and *I. difengpi*; Table S2: The codons of *I. verum* and *I. difengpi*; Table S3: The SSRs of *I. verum* and *I. difengpi*.

## Abbreviations

The following abbreviations are used in this manuscript:

cp	Chloroplast
IRs	Inverted repeats
LSC	Large-single-copy
SSC	Small-single-copy
tRNA	Transfer RNA
rRNA	Ribosome RNA
Nr	Non-redundant
COGs	Clusters of Orthologous Groups
KEGG	Kyoto Encyclopedia of Genes and Genomes
GO	Gene Ontology
RSCU	Relative synonymous codon usage
MISA	MIcroSAtellite
ML	Maximum likelihood
SSRs	Simple sequence repeats
ML BSs	ML bootstrap support values

## References

[1] Bremer B., Bremer K., Chase M., Reveal J.L., Soltis P., Stevens P., Anderberg A., Fay M., Goldblatt P., Judd W. (2003). An update of the Angiosperm Phylogeny Group classification for the orders and families of flowering plants: APG II. Bot. J. Linn. Soc..

[2] Iii A., Bremer K. (2009). Angiosperm Phylogeny Group III (APG III). An update of The Angiosperm Phylogeny Group classification for the orders and families of flowering plants: APG III. Botanical Journal of the Linnean Society. Bot. J. Linn. Soc..

[3] Zou Q., Huang Y., Zhang W., Lu C., Yuan J. (2023). A Comprehensive Review of the Pharmacology, Chemistry, Traditional Uses and Quality Control of Star Anise (*Illicium verum* Hook. F.): An Aromatic Medicinal Plant. Molecules.

[4] Shahrajabian M., Sun W., Cheng Q. (2020). Chinese star anise (*Illicium verum*) and pyrethrum (*Chrysanthemum cinerariifolium*) as natural alternatives for organic farming and health care- A review. Aust. J. Crop Sci..

[5] Wu C., Liang H., Qi B., Liu B., Wang M., Tang H., Li D., Fahad S. (2021). Research progress on *Illicium difengpi* (Illiciaceae): A review. Horticulturae.

[6] Zhu L., Luo Y., Xiao J., Hao E., Wei J., Zhao J., Yao C., Wang Y., Luo H. (2024). Star anise (*Illicium verum* Hook. f.): Dual therapeutic and nutritional potential in food and medicine. Acupunct. Herb. Med..

[7] Ning D.S., Fu Y.X., Peng L.Y., Tang H., Li L.C., Wu X.D., Huang Y.S., Pan Z.H. (2020). Phytochemical constituents of the pericarps of *Illicium difengpi* and their anti-inflammatory activity. Nat. Prod. Res..

[8] Pan Z.-H., Cheng L., Ning D.-S., Peng L.-Y., Fu Y.-X., Li L.-C. (2019). Difengpienols A and B, two new sesqui-neolignans with anti-inflammatory activity from the bark of *Illicium difengpi*. Phytochem. Lett..

[9] Huang J., Liu H., Yang C., Ye J., Xue Y. (2000). Morphological identification of fruits from 16 illicium species. Chin. Tradit. Herb. Drugs.

[10] Wen S. (1990). Comparison study of volatile oil content and toxicity test between *Illicium verum* and its adulterating species. China J. Chin. Mater. Medica.

[11] Mower J.P., Vickrey T.L., Chaw S.-M., Jansen R.K. (2018). Chapter Nine—Structural Diversity Among Plastid Genomes of Land Plants. Advances in Botanical Research.

[12] Shaw J., Lickey E.B., Schilling E.E., Small R.L. (2007). Comparison of whole chloroplast genome sequences to choose noncoding regions for phylogenetic studies in angiosperms: The tortoise and the hare III. Am. J. Bot..

[13] Cao Y., Lai Y., Li Z., Zhai S., Dai Y., Tao J., Wang Q., Xu Z., Jiang M., Yu L. (2024). The complete chloroplast genome of *Illicium verum* and comparative analysis with related species from Magnoliaceae and Illiciaceae. Front. Genet..

[14] Park J., Kim Y., Xi H. (2019). The complete chloroplast genome of aniseed tree, *Illicium anisatum* L. (Schisandraceae). Mitochondrial DNA Part B.

[15] Zhou F., Liu Y., Xiong S., Huang Y. (2024). The complete chloroplast genome of *Illicium simonsii* Maxim. (Illiciaceae), a species with important medicinal properties. Mitochondrial DNA Part B.

[16] Hansen D.R., Dastidar S.G., Cai Z., Penaflor C., Kuehl J.V., Boore J.L., Jansen R.K. (2007). Phylogenetic and evolutionary implications of complete chloroplast genome sequences of four early-diverging angiosperms: *Buxus* (Buxaceae), *Chloranthus* (Chloranthaceae), *Dioscorea* (Dioscoreaceae), and *Illicium* (Schisandraceae). Mol. Phylogenetics Evol..

[17] MeiZi L., Hui Y., Kun L., Pei M., Wenbin Z., Ping L. (2012). Authentication of *Illicium verum* using a DNA barcode *psbA-trnH*. J. Med. Plants Res..

[18] Zhou Q.-R., Ma Y.-Y., Lv H.-Q., Lu Z.-C., Wang L.-S., Liang J.-S., Li J.-J. (2024). Chloroplast mini-barcodes combined with high resolution melting analysis to identify herbal medicine Difengpi (*Illicium difengpi*). Heliyon.

[19] Li B., Zheng Y. (2018). Dynamic evolution and phylogenomic analysis of the chloroplast genome in Schisandraceae. Sci. Rep..

[20] Morris A.B., Bell C.D., Clayton J.W., Judd W.S., Soltis D.E., Soltis P.S. (2007). Phylogeny and Divergence time estimation in *Illicium* with implications for New World biogeography. Syst. Bot..

[21] White D.A., Leonard B.T. (1985). The pollination of *Illicium parviflorum* (Illiciaceae). J. Elisha Mitchell Sci. Soc..

[22] Roberts M.L., Robert R.H. (1983). Ballistic seed dispersal in *Illicium* (Illiciaceae). Plant Syst. Evol..

[23] Zhang J., Chen M., Dong X., Lin R., Fan J., Chen Z. (2015). Evaluation of four commonly used DNA barcoding Loci for chinese medicinal plants of the family schisandraceae. PLoS ONE.

