# Supplementary material for: A Comparative Analysis and Phylogenetic Relationship of the Chloroplast Genome Sequences of Illicium verum and Illicium difengpi
Source: Genes (Basel). 2025 Mar 8;16(3):321. doi: 10.3390/genes16030321 (PMC11942347; doi:10.3390/genes16030321)
Supplement: Supplementary file 1 [file genes-16-00321-s001.zip › genes-3510019-supplementary.pdf]

A comparative analysis and phylogenetic relationship of the chloroplast genomes sequences of *Illicium verum* and *Illicium difengpi*

Suqin Guo [1], Xiqun Wu [1], Feng Peng [2], Kun Zhang [2], Suren Sooranna [3], Guiyu Tan [2,*]

1 School of Pharmacy and Food Engineering, Wuyi University, Jiangmen, Guangdong, 529020, China; guosuqin074910@163.com,
2 Key Laboratory of High-quality Formation and Utilization of Dao-di Herbs, Guangxi Botanical Garden of Medicinal Plants, Nanning 530023, China
3 Department of Metabolism, Digestion and Reproduction, Faculty of Medicine, Imperial college London, London, SW10 9NH, UK

* Correspondence: tangy@gxyyzwy.com

Abstract: Background/Objectives: **Background/Objectives**: *Illicium verum* Hook. f. and *I. difengpi* K. I. B.et K. I. M., are two unique plants which grow in the mountainous areas of Guangxi, China. Their similar morphological characteristics frequently lead to their misidentification. Chloroplast genome (cp)-based barcode technology has been used to effectively identify two closely related species, although at present, there is no systematic comparative study of the cp genome sequences between *I. verum* and *I. difengpi*. **Methods**: Here, the cp genomes of the two species were sequenced and analyzed. **Results**: The cp genome sizes were 142,689 and 142,689 bp for *I. verum* and *I. difengpi,* respectively. Each of the cp genomes annotated 122 genes, including 79 protein coding genes, 8 ribosomal RNA genes, and 35 transfer RNA genes. Amino acid frequencies of 1.17-10.19% (*I. verum*) and 1.18-10.17% (*I. difengpi*) were found in the cp genome protein coding genes. There were also 104 and 96 SSRs as well as 26 and 25 long repeats identified in the cp genome of *I. verum* and *I. difengpi*, respectively, among which the most common were A/T base repeats. Both cp genomes had SSC/IRa junctions located in gene *ycf1-trnN.* The *ycf1* and *trnL-trnV-rps7* genes were located at the IRb/SSC and LSC/IR boundaries, respectively. **Conclusions**: A phylogenetic relationship was constructed, and the two species were fully nested within the genus, *Illicium*. The comparative cp genomes of *I. verum* and *I. difengpi* are presented in this study, and this provides valuable phylogenetic information for subsequent molecular marker development and research of *I. verum* and *I. difengpi*.

Keywords: *Illicium verum*; *Illicium difengpi*; chloroplast genome; phylogenetic relationship

Table S1 Characteristics of the complete chloroplast genomes of *I. verum* and *I. difengpi*.

Species		*I. verum*	*I. difengpi*
Length (bp)	All	142,689	143,629
	LSC	101,100	101,450
	SSC	19,689	20,237
	IR	10,949	10,971
GC (%)	Total	39.13	39.10
	LSC	37.96	37.98
	SSC	34.16	33.91
	IR	49.06	49.06
Gene number	Total	122	122
	Protein-coding gene	79	79
	rRNA gene	8	8
	tRNA gene	35	35

Table S2 The codons of *I. verum* and *I. difengpi*.

Species		*Illicium verum*			*Illicium difengpi*		
**Amino Acid**	**Codon**	**Number**	**Frequency**	**RSCU**	**Number**	**Frequency**	**RSCU**
Ala	GCA	362	1.58	1.11	360	1.57	1.10
	GCC	223	0.97	0.69	222	0.97	0.68
	GCG	140	0.61	0.43	140	0.61	0.43
	GCT	575	2.51	1.77	582	2.53	1.79
Arg	AGA	441	1.92	1.86	445	1.94	1.87
	AGG	172	0.75	0.73	174	0.76	0.73
	CGA	297	1.30	1.25	297	1.29	1.25
	CGC	97	0.42	0.41	100	0.44	0.42
	CGG	99	0.43	0.42	99	0.43	0.42
	CGT	316	1.38	1.33	312	1.36	1.31
Asn	AAC	247	1.08	0.47	251	1.09	0.48
	AAT	796	3.47	1.53	803	3.50	1.52
Asp	GAC	200	0.87	0.43	195	0.85	0.42
	GAT	726	3.17	1.57	727	3.17	1.58
Cys	TGC	72	0.31	0.54	74	0.32	0.55
	TGT	196	0.85	1.46	197	0.86	1.45
Gln	CAA	585	2.55	1.47	585	2.55	1.46
	CAG	211	0.92	0.53	215	0.94	0.54
Glu	GAA	875	3.82	1.47	879	3.83	1.47
	GAG	319	1.39	0.53	321	1.40	0.54
Gly	GGA	639	2.79	1.56	637	2.77	1.56
	GGC	167	0.73	0.41	170	0.74	0.42
	GGG	280	1.22	0.68	279	1.21	0.68
	GGT	550	2.40	1.34	552	2.40	1.35
His	CAC	142	0.62	0.47	144	0.63	0.48
	CAT	456	1.99	1.53	453	1.97	1.52
Ile	ATA	628	2.74	0.96	630	2.74	0.96
	ATC	433	1.89	0.66	434	1.89	0.66
	ATT	906	3.95	1.38	900	3.92	1.37
Leu	CTA	321	1.40	0.83	322	1.40	0.83
	CTC	168	0.73	0.43	171	0.74	0.44

	CTG	189	0.82	0.49	192	0.84	0.49
	CTT	474	2.07	1.22	471	2.05	1.21
	TTA	672	2.93	1.73	672	2.93	1.73
	TTG	504	2.20	1.30	507	2.21	1.30
Lys	AAA	802	3.50	1.45	804	3.50	1.44
	AAG	303	1.32	0.55	309	1.35	0.56
Met	ATG	568	2.48	1.00	565	2.46	1.00
Phe	TTC	512	2.23	0.83	514	2.24	0.83
	TTT	725	3.16	1.17	718	3.13	1.17
Pro	CCA	287	1.25	1.17	285	1.24	1.16
	CCC	220	0.96	0.90	221	0.96	0.90
	CCG	109	0.48	0.44	110	0.48	0.45
	CCT	366	1.60	1.49	366	1.59	1.49
Ser	AGC	111	0.48	0.38	110	0.48	0.37
	AGT	350	1.53	1.19	348	1.52	1.18
	TCA	381	1.66	1.29	384	1.67	1.30
	TCC	299	1.30	1.01	299	1.30	1.01
	TCG	152	0.66	0.52	152	0.66	0.52
	TCT	475	2.07	1.61	476	2.07	1.61
Thr	ACA	366	1.60	1.25	368	1.60	1.26
	ACC	217	0.95	0.74	215	0.94	0.74
	ACG	126	0.55	0.43	127	0.55	0.43
	ACT	461	2.01	1.58	460	2.00	1.57
Trp	TGG	407	1.78	1.00	406	1.77	1.00
Tyr	TAC	178	0.78	0.42	179	0.78	0.42
	TAT	664	2.90	1.58	668	2.91	1.58
Val	GTA	456	1.99	1.41	454	1.98	1.41
	GTC	170	0.74	0.53	170	0.74	0.53
	GTG	206	0.90	0.64	206	0.90	0.64
	GTT	462	2.01	1.43	461	2.01	1.43

Table S3 The SSRs of *I. verum* and *I. difengpi*.

Repeat unit	Repeat number	Size	Number of *I. verum*	Number *I. difengpi*
A	10	10	8	5
A	11	11	7	7
A	12	12	4	5
A	14	14	2	3
A	15	15	1	2
A	16	16	0	1
A	17	17	1	0
A	19	19	1	0
C	10	10	2	0
T	10	10	10	8
T	11	11	4	8
T	12	12	3	2
T	13	13	2	2
T	14	14	0	2
T	15	15	1	0
T	16	16	1	0
T	18	18	1	1
AG	5	10	1	0
AT	5	10	1	1
AT	6	10	1	1
TA	5	10	1	1
TC	5	10	2	2
AAT	4	12	1	0
ACC	4	12	1	1
TAT	4	12	1	1
TTC	4	12	0	1
AAAT	3	12	1	1
AATG	3	12	1	1
ATTT	3	12	1	1
CATT	3	12	1	1
TTCT	3	12	1	1
TATC	4	16	0	1
AACTA	3	15	1	1
TTCAT	3	15	1	0
CGTTAA	3	18	0	1
TTAACG	3	18	0	1
Others	\	\	19	15
Total number	\	\	83	78
